# Tuberculosis Care Access Among Internally Displaced Persons in India and Nigeria: A Review of Structural Barriers and Context‐Specific Interventions

**DOI:** 10.1002/puh2.70319

**Published:** 2026-07-23

**Authors:** Paul West Okojie, Linnaya Graf

**Affiliations:** ^1^ Liberty University Lynchburg Virginia USA

**Keywords:** healthcare access, India and Nigeria, internally displaced persons, intervention strategies, structural barriers, tuberculosis care

## Abstract

This review examined the structural barriers limiting access to tuberculosis (TB) care in internally displaced persons and context‐specific strategies to address the public health threat. Low‐ and middle‐income countries contribute more than 95% of the global burden of tuberculosis, with Nigeria and India being the highest TB‐burdened countries in Africa and Asia, respectively. Despite this burden, internally displaced persons remain underrepresented in TB research. Both countries share similarities in demographic, economic, and sociopolitical backgrounds that raise the risk of TB spread among vulnerable populations, including IDPs. Communal conflict, disasters, and insecurity promote internal population displacement, but India's share of global IDPs is mainly due to climate events and natural disasters. In both countries, sociocultural, economic, and healthcare governance concerns remain significant barriers to TB care access among IDPs. Although the health of all people is a fundamental human rights issue, selective recognition of those rights to the detriment of the marginalized group perpetuates health inequities. Unlike Nigeria, India lacks an actionable framework for protecting the health of internally displaced persons. The review identified that TB control and access to TB care efforts require intersectoral collaboration to help fast‐track movement toward TB elimination. It further underscores the relevance of primary healthcare infrastructure tailored to local contexts. It concludes by recommending prioritizing policy reforms that promote equity in TB care, with an expansion of TB interventions to address the threat among internally displaced persons.

## 1 Introduction

Despite efforts, tuberculosis (TB) remains a global threat to public health. Nigeria and India are highly TB‐burdened countries in Asia and Africa, respectively. India is the most TB‐burdened country in the world, contributing over 2 million cases annually to the global burden of TB. Nigeria reports about 500,000 new TB cases, the highest in Africa, and accounts for nearly 4% of the global TB incidence [1, 2]. From 2000 to 2021, Nigeria recorded an increase in reported TB cases from 269,000 to 467,000 [1]. Within the same period, Nigeria experienced an estimated 268 people dying daily from the disease and many cases unreported [3]. In 2023, the country recorded 403,000 cases and 157,000 TB‐related deaths [1]. Nigeria and India are part of low‐ and middle‐income countries. Both countries account for nearly 95% of TB‐related deaths globally [3]. TB affects people of all age groups, races, and ethnicities, but the risk is unequal even within the same country or community. The disease disproportionately affects people in poor and adverse living conditions, including the internally displaced population. This has implications for TB prevention and control strategies, which must be designed to address the social, economic, and cultural realities of affected individuals and communities.

This review focuses on Nigeria and India as case countries because they share several significant similarities, particularly in their colonial history, diversity, and democratic structures [4]. Both countries are former British colonies with ethnically and culturally diverse populations. Nigeria has over 200 languages and 250 ethnic groups, whereas India has over 200 distinct languages and multiple ethnic groups, reflecting their complex ethnocultural diversity [4]. Although India is an emerging economy, Nigeria is the continent's third‐largest economy, with the prospect of joining the ranks of other emerging economies. Both countries have large agricultural sectors and vast rural populations. Developmentally, both countries are considered less developed, face infrastructural challenges, and have significantly impoverished populations in both urban and rural communities. This review is significant because it synthesizes fragmented evidence on TB care among internally displaced persons, a population that is underrepresented in public health research. The review further identifies social, structural, and political barriers and highlights context‐specific intervention strategies. Most reviews of TB care focus either on a single country or on the challenges faced by different vulnerable groups, without a cross‐regional comparison of internally displaced persons and their TB care access barriers. Comparing India and Nigeria provides insights for policymakers, health program planners, and humanitarian actors seeking to improve TB diagnosis, treatment adherence, and health outcomes in these highly vulnerable populations, while also identifying gaps for future research. Therefore, this article will examine the structural challenges to accessing TB care among internally displaced persons in India and Nigeria and the interventions to address them.

### 1.1 Demographic Profile of India and Nigeria

Nigeria is home to over 220 million people and has an annual population growth rate of 2.35% [1]. It is demographically categorized as a young population, with a median age of 18.1 years, and over 60% of the population is under 24 years old. Similarly, India has a broad‐based population pyramid, reflecting its young population. It has a population of 1.4 billion people (the highest in the world) and a median age of 28.8 years [5]. Although slightly more than 50% of Nigeria's population resides in rural areas, over 60% of the Indian population resides in urban centers [6, 7]. Poverty is prevalent in both countries, with the populations characterized by low socioeconomic circumstances, exacerbated by limited education and a lack of social amenities, which negatively impact their health outcomes [8, 9, 10].

### 1.2 Internally Displaced Persons in India and Nigeria

Internal crises, such as insurgency, ethnic clashes, and disasters, lead to population displacement. Since 2009, millions have been displaced in Nigeria due to security challenges by armed groups and terrorists, including violent ethnic and communal clashes [8]. Unlike in Nigeria, most population displacement in India is due to natural disasters, development‐induced, and ethnic clashes [11]. Data from the European Union, the United Nations, and other international organizations, such as the United Nations Statistical Commission and the International Organization for Migration (IOM), complements national data sources on the demographic characteristics of marginalized groups, including IDPs in Nigeria [12]. At the end of 2023, there were an estimated 3.3 million internally displaced persons in Nigeria [12]. An IOM report on IDPs in eight states across northwest and north central Nigeria revealed that, at the end of 2023, there were 1,092,196 internally displaced persons in both subregions [13]. The IOM data Atlas showed that 54% of IDPs in both subregions were males, whereas 46% were females. According to the IOM report, 54% of the IDPs were minors or under 18 years, whereas 17% of the IDPs were aged 60 years or more, which reflects the country's young demographic structure [13]. The above data are similar to those of Nigeria's National Bureau of Statistics 2024 report on internally displaced persons, which put the total number of IDPs in Nigeria at 1,134,828, with 50.3% of them being below the age of 18 years, 50.5% being males, and 49.5% being females [14].

There were 8.2 million internally displaced persons in Southeast Asia in 2022 [15]. India recorded 5.4 million displacements in 2024, including 2.4 million triggered by severe monsoon floods [16]. At the end of 2024, there were 643,000 internally displaced persons in India [16]. Despite India's much larger population, Nigeria had a susbtantially larger internally displaced persons at the end of 2024 (approximatetely 3.7 million versus 643,000, respectively) [16]. Although data on the age and sex profile of IDPs in Nigeria are available, disaggregated data on IDPs by sex in India are limited. A 2022 study addressed the gender perspective of key development challenges around IDPs, in which it was shown that India was not among the countries with sex‐disaggregated IDP data [17]. This poses a limitation to research inquiring into the sex ratio of IDPs in the country, how they are impacted by economic, environmental, and social factors, and is a potential barrier to holistic planning to address the health and other needs of internally displaced persons in India.

### 1.3 Internally Displaced Persons and TB Risk

Crisis‐induced population displacement dislocates people from their functional social infrastructure, if it is available at all. Most of these internally displaced persons are sheltered in camps with less‐than‐ideal conditions characterized by a lack of basic sanitation and hygiene facilities [18, 19]. Poor nutrition, overcrowding, and preexisting medical conditions like diabetes and HIV, including a lack of TB screening and monitoring, increase the risk of TB outbreaks, making IDPs vulnerable to the disease [20, 21, 22, 23]. A study in Northeast Nigeria on TB and HIV outreach services to internally displaced populations tested 17,000 IDPs from three states for TB [8]. It found a TB burden more than twice the estimated national TB incidence and 10 times the national notification rate [8]. These findings signaled the scale of the burden of TB among displaced populations and the need to expand interventions to curb the public health threat.

## 2 Methods

### 2.1 Literature Search Strategy

A narrative review approach was used to integrate evidence across disciplines, namely, public health, geography, human rights, and demography. This interdisciplinary synthesis highlights TB as a marker of social marginalization and structural disadvantage. This review approach was selected because it enabled the exploration of the broad range of structural determinants influencing TB care access among IDPs rather than evaluating the effectiveness of a specific intervention. Given the heterogeneity and the limited number of IDP‐specific studies, this approach enabled a comprehensive understanding of the structural barriers and context‐specific interventions affecting TB care access among internally displaced populations in India and Nigeria.

A comprehensive literature search was conducted to identify peer‐reviewed empirical studies and authoritative grey literature on TB care access among internally displaced persons (IDPs) in India and Nigeria. The search focused on structural barriers to TB care access and context‐specific interventions in both countries. The electronic database search covered PubMed, Scopus, Global Index Medicus (GIM), and Google Scholar. To supplement this, authoritative governmental and public health organization sources were searched for relevant literature. Grey literature was identified through searches of publications and repositories from the World Health Organization (WHO), United Nations High Commission for Refugees (UNHCR), IOM, ReliefWeb, Stop TB Partnership, National Tuberculosis Elimination Program (NTEP) India, and National Tuberculosis, Leprosy and Buruli Ulcer Control Programme (NTBLCP) Nigeria.

### 2.2 Search Framework

The search strategy was structured using five concept domains combined with Boolean operators: (1) TB (including drug‐resistant TB), (2) internal displacement and conflict‐affected populations (e.g., IDPs, forced migration, humanitarian settings, and refugees), (3) access to care outcomes (e.g., utilization, coverage, adherence, treatment outcomes, case detection, and loss to follow‐up), (4) structural barriers and interventions (e.g., health system constraints and programmatic strategies), and (5) geographic focus (India OR Nigeria, including conflict‐affected regions such as Borno, Adamawa, Yobe, Jammu and Kashmir, Manipur, Assam, and Chhattisgarh). Within each concept domain, synonyms and related terms were combined using OR, and concepts were combined using AND. The detailed database‐specific search strings applied are shown in Table S1.

#### 2.2.1 Inclusion Criteria

Studies were included using the following criteria: addressed TB (all forms), focused on characteristics of IDP/IDP camps in India or Nigeria, and TB risk in internally displaced populations. Additional criteria for inclusion were studies that examined conflict‐affected populations or closely related structurally vulnerable groups and addressed TB access to care, barriers, or interventions. Moreover, studies were included if they were conducted in or directly relevant to India or Nigeria, and published between 2015 and 2025, and were available in English.

#### 2.2.2 Exclusion Criteria

The following exclusion criteria were used: They did not address displacement or conflict‐affected populations, focused solely on clinical, microbiological, or laboratory outcomes without access or systems relevance, were opinion pieces, editorials, or letters without empirical or analytical content, were duplicate reports of the same dataset (most complete version retained), or focused exclusively on migrant/refugee populations outside India or Nigeria.

### 2.3 Data Extraction and Analysis

Records from the databases were downloaded and uploaded to Endnote for title abstract screening. Predefined inclusion and exclusion criteria were used to evaluate all selected abstracts. Summary information was extracted using an Excel data extraction table. The extracted data included domains of challenge to TB care access in India and Nigeria, and key insights were summarized comparatively.

## 3 Results

**FIGURE 1:**
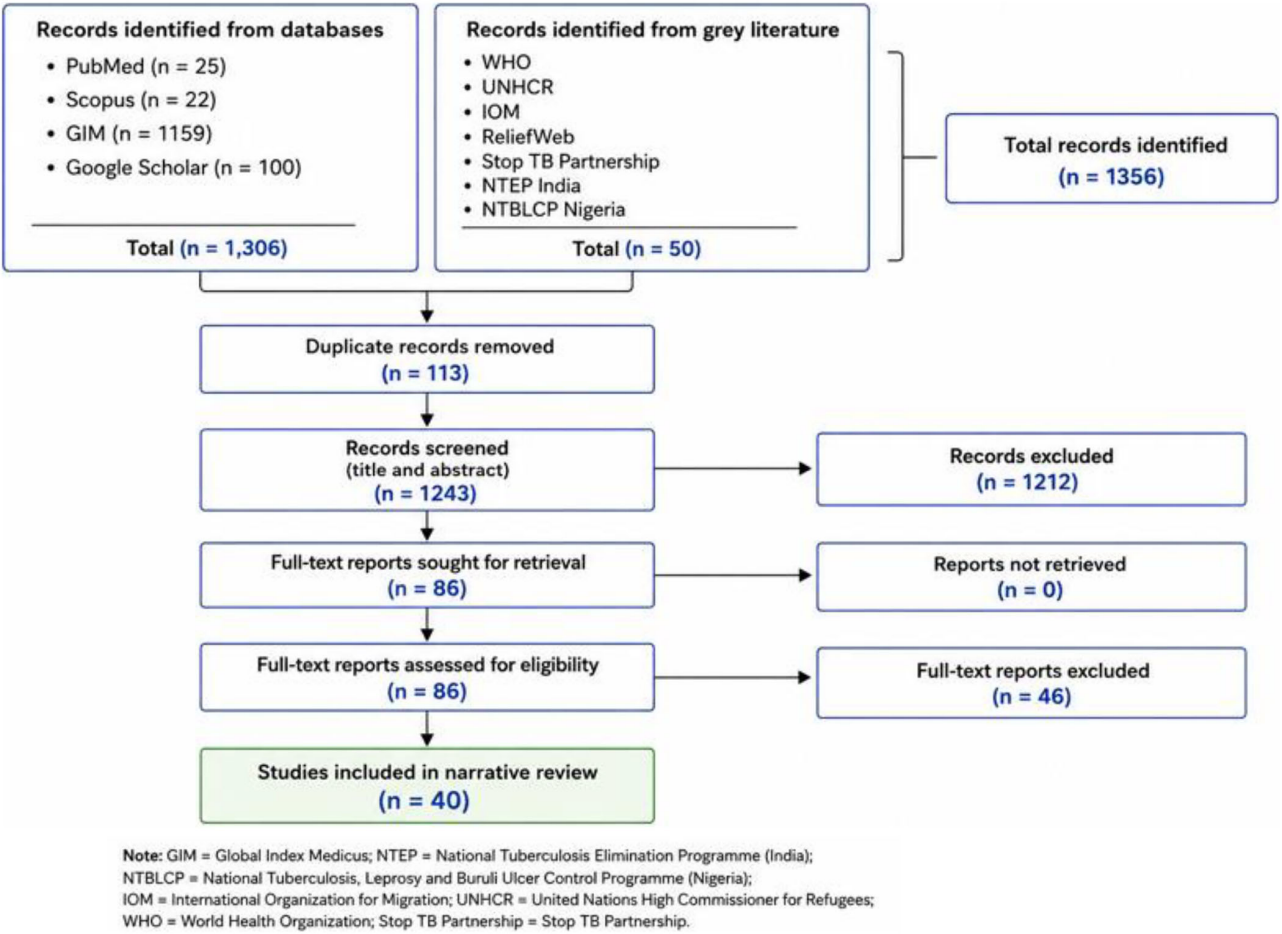
Flowchart of the study selection procedure. TB, tuberculosis.

A total of 1356 records were identified in the electronic databases before deduplication. After removing duplicate records, 1243 unique records remained and were screened based on title and abstract. During this stage, 1212 records were excluded for not meeting the eligibility criteria, leaving 86 reports for full‐text retrieval and review. All 86 reports were successfully retrieved and assessed for eligibility. Following full‐text evaluation, 40 reports were included in the narrative review. A summary of the selection process is presented in Figure 1. The included literature captured peer‐reviewed articles and documents on TB epidemiology, prevention, and control, as well as authoritative reviews and reports on IDPs in India and Nigeria. Literature reporting on cross‐border displacement from natural disasters and conflict with no relevance to IDPs was excluded.

### 3.1 Analytic Approach

This review employed a comparative analysis framework to organize findings thematically across key domains from the literature review. Selected literature was used to identify themes related to barriers to TB care access, including sociocultural, economic, and health governance, policy, and human rights aspects in India and Nigeria. Similarly, literature was used to identify country‐specific strategies that addressed the thematic barriers. Key comparisons from the literature are summarized in Table 1.

**TABLE 1:** Comparative barriers and intervention strategies for tuberculosis (TB) care access among internally displaced persons (IDPs) in Nigeria and India.

Domain	Nigeria	India	Key comparative insight
**Sociocultural** *Gender roles in health‐seeking behavior*	Strong patriarchal norms limit women's autonomy, leading to delayed or nonuse of TB services	Patriarchal norms also limit women's decision‐making autonomy, leading to delayed care‐seeking	Both countries exhibit gender inequality affecting TB access. No major difference; India has more structured gender‐responsive TB programming
*Cultural beliefs, stigma and practices*	Reliance on traditional healers; myths and stigma lead to delayed treatment	Similar reliance on traditional healers; stigma and misinformation lead to delayed care	Strong similarity in stigma and cultural beliefs affecting TB care. No major difference
**Economic** *Out‐of‐pocket expenditure*	Indirect costs (transport, food, lost income) remain major barriers despite free TB services	High out‐of‐pocket expenditure (∼63% of health spending) creates major financial burden	Both face significant financial barriers to TB care. India has structurally higher national OOP burden
*Poverty*	High multidimensional poverty (∼63%) increases TB vulnerability and limits access	Large poor population (∼269 million) with strong TB–poverty link	Poverty strongly drives TB risk in both countries. India historically has larger absolute poor population and more quantified TB‐poverty data
**Health governance** *Policy and infrastructure*	National TB Strategic Plan exists but is weakly implemented due to funding and human resource gaps	NTEP exists but rural infrastructure and implementation gaps remain	Both have TB programs with implementation challenges. India shows more advanced program reform and structure
*Leadership and systems*	Limited coordination and weak integration of IDP TB needs	Strong political commitment, decentralization, and digital TB systems	TB prioritized in both countries. India demonstrates stronger governance and innovation
**Legal and human rights** *IDP frameworks*	Nigeria has IDP frameworks (NCFRMI, disaster policy) but weak implementation	No comprehensive IDP legal framework; limited UNHCR access	Both have gaps in operationalizing human rights for IDPs. Nigeria has more formal IDP institutional structures
**Social determinants of health**	IDPs face poor housing, malnutrition, and limited healthcare access	Similar deprivation among poor and displaced populations	Strong SDH influence on TB vulnerability in both. Nigeria IDP conditions described as more severe
**Interventions** *Sociocultural*	Limited structured TB stigma/gender interventions for IDPs	Gender‐Responsive TB Framework and Action Against Stigma initiative	Both recognize stigma as a barrier. India has more formalized national interventions
*Economic support*	No structured TB cash transfer or nutrition support system described	Ni‐kshay Poshan Yojana provides monthly cash transfer for TB patients	Both aim to reduce financial barriers. India has formal social protection for TB patients
*Governance and innovation*	Limited TB digital or innovation systems for IDPs	Digital surveillance, AI tools, decentralization, and community engagement under NTEP	Both operate national TB programs. India shows stronger technological and system innovation

Abbreviations: NCFRMI, National Commission for Refugees, Migrants, and Internally Displaced Persons; NTEP, National Tuberculosis Elimination Program; TB, tuberculosis.

### 3.2 Barriers to TB Care Access by Internally Displaced Persons

**FIGURE 2:**
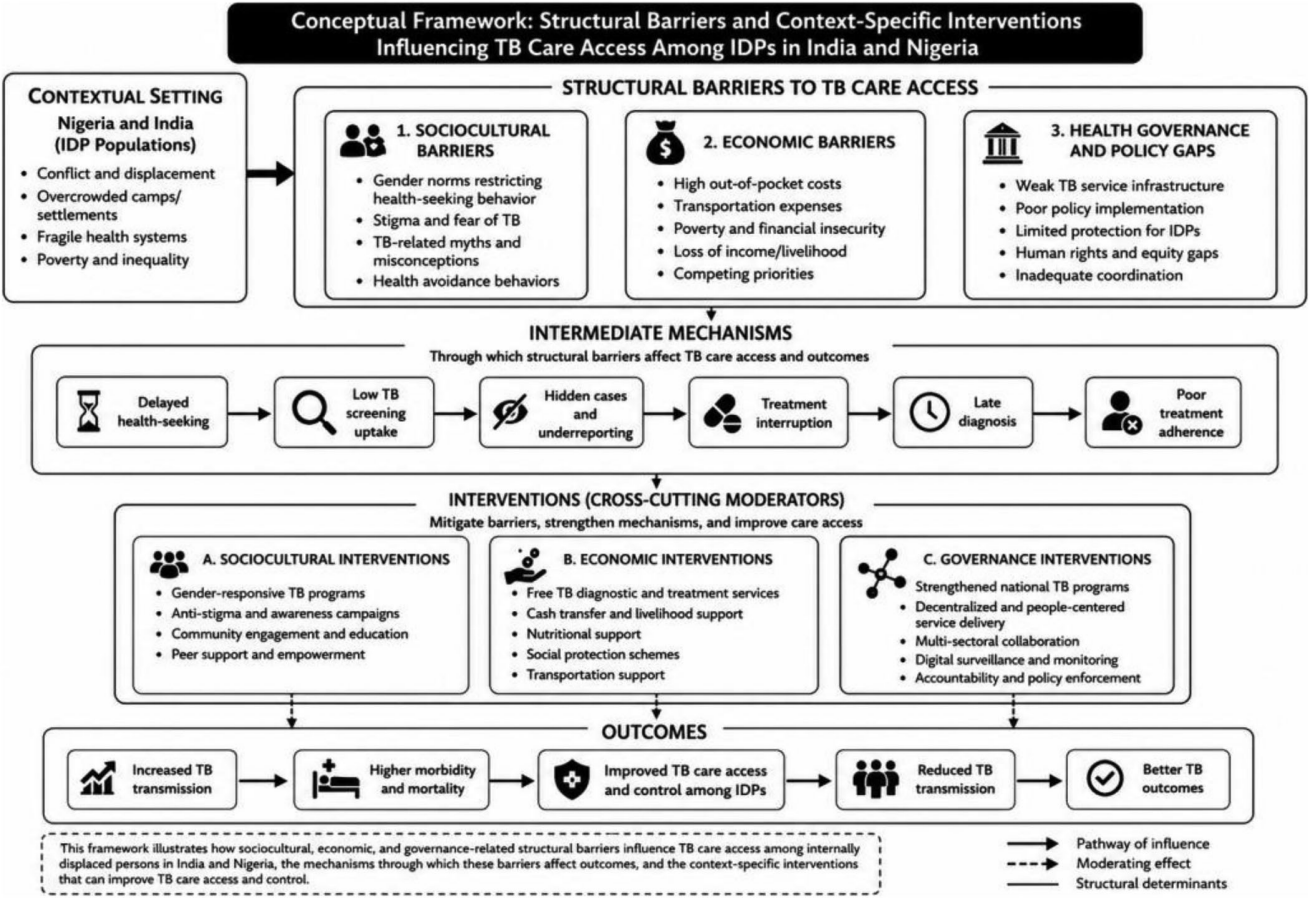
Conceptual diagram of contextual barriers to TB care access and interventions among IDPs: India versus Nigeria. TB, tuberculosis.

This review revealed that there are similarities and subtle differences between Nigeria and India in terms of factors challenging the access of IDPs to TB care. Figure 2 presents a conceptual framework illustrating structural barriers to, and interventions for, TB care access among IDPs in India and Nigeria.

#### 3.2.1 Sociocultural

##### 3.2.1.1 Gender Roles in Health‐Seeking Behavior

India and Nigeria are both paternalistic, male‐dominated societies, and most women cannot decide on their health without a man's permission. Studies have shown that gender imbalance due to cultural norms can negatively affect TB care and service utilization and outcomes through needless delays or refusal to seek care [24]. A prior study examining women and TB care in India revealed that women have limited autonomy in making decisions about their health [25]. This traditional or cultural notion constitutes a barrier to the acceptance or uptake of TB treatment and prevention services [2].

##### 3.2.1.2 Cultural Beliefs and Practices

Like Nigeria, most IDPs in India come from communities with limited access to formal healthcare, they rely heavily on traditional healers whose approaches to care are rooted in cultural and traditional beliefs [26, 27, 28]. The myths and misconceptions about the causes and effects of TB are similar between rural India and the Nigerian population. Most people living with TB (PLwT) live in fear of being stigmatized, shunned, or forced to quit their jobs [24, 28]. Studies further show that due to a “lack of knowledge about the disease and fear of being ostracized, people with TB often hide their symptoms and fail to receive appropriate treatment” [24], a major challenge to TB treatment, prevention, and control.

#### 3.2.2 Economic

##### 3.2.2.1 Cost of Healthcare/Out‐of‐Pocket (OOP) Spending

The availability of free diagnosis and treatment for TB cases in Nigeria does not preclude OOP healthcare expenditures, which can be prohibitively expensive. Other indirect costs associated with healthcare, such as transportation, food, and time lost traveling to seek care, are significant burdens on the impoverished rural and urban populations. The situation is not markedly different in India, where OOP spending on healthcare accounts for nearly 63% of total health expenditure, one of the highest globally. The enormous financial implication of OOP spending and the opportunity cost constitute a barrier to accessing TB care by vulnerable populations such as migrant workers, including internally displaced persons [29, 30].

##### 3.2.2.2 Poverty and Limited Income Sources

There is a historical and scientific correlation between poverty and TB [31, 32]. The impact of poverty on the urban and rural poor in Nigeria and India is not remarkably different [33]. A report showed that there is a higher prevalence of TB among the multidimensionally poor compared to the non‐multidimensionally poor in India [31]. Based on the methodology of the Suresh Tendulkar Committee report of 2009, nealy 269 million Indians were below the poverty line [34]. Similarly, about 63% of the Nigerian populations (133 million) are multidimensionally poor; in other words, they experience deprivation in more than one dimension [35]. Poverty creates hardships and worsens living conditions, and many IDPs in both India and Nigeria are unable to meet their basic health needs. This poor economic outlook increases individual and community vulnerability to TB and limits their access to care.

#### 3.2.3 Health Governance, Policy, and Human Rights

##### 3.2.3.1 Inadequate Policy Implementation, Funding, and Infrastructural Issues

Nigeria has a National Strategic Plan for TB, whose implementation is hindered by funding constraints, inadequate human resources, and a weak leadership structure [1]. Similarly, India's response to TB is affected by poor healthcare infrastructure, particularly in primary healthcare facilities in rural areas, and a gap in TB control policy implementation [36, 37].

##### 3.2.3.2 Legal, Policy, and Human Rights

Inequities significantly contribute to the global burden of TB [38]. For the displaced population, their rights to legal protection and advocacy for optimal health and protection are challenged by a lack of significant attention from authorities, which excludes them from existing health programs [38]. The United Nations’ Universal Declaration of Human Rights recognizes the fundamental importance of upholding the dignity and equal and inalienable rights of all members of the human family as the foundation for global freedom, peace, and justice, including the right of all people to health [39]. The promotion of human rights, related to the social determinants of health, such as the right to food and nutrition, the right to shelter, and the right to education, reduces vulnerability to infectious diseases such as TB. According to the UN's guiding principles on internal displacement, displaced people have a right to basic humanitarian assistance, including food, medicine, and shelter [40]. They also have a right to education, protection from violence, and the right to participate in economic activities [40].

In Nigeria's northeast, IDPs live in poor housing conditions, lack proper nutrition, and, in most cases, lack access to health information and services needed to prevent, control, and manage the disease [8]. The inadequate presence of these social determinants of health inevitably creates health equity concerns, resulting from the poor health status of the displaced population compared to those living in relatively ideal conditions. Despite the role of social determinants of health in achieving health equity, a comprehensive primary healthcare system is also important because it provides essential healthcare that is universally accessible, acceptable, available, and with the use of appropriate technologies that are consistent with the level of development of the country at an affordable cost to individuals and families in the community [41]. The lack of access to essential primary healthcare services by displaced persons in Nigeria's northeast is a human rights issue because it widens the health equity gap between displaced persons and other members of the population. Though both Nigeria and India have national TB response guidelines and frameworks, unlike Nigeria, India has no national policy or legal institutional framework to address the issues of refugees or internally displaced persons. India has not ratified the 1951 Convention and 1967 Protocol, nor does it permit the United Nations High Commission for Human Rights (UNHCR) access to most of the country's refugee populations. India also lacks a national policy on resettlement and rehabilitation of internally displaced persons who are at risk of contracting TB [42]. An attempt at addressing the plight of the Indian IDPs was the “draft national policy for rehabilitation of persons displaced as a consequence of the acquisition of land” [42]. But this draft policy completely disregards the plight of IDPs of other categories, including those fleeing human rights violations, physical violence, and communal and other sources of tension [42]. Although India lacks a comprehensive national policy or dedicated institutional framework for the resettlement and rehabilitation of internally displaced persons [42], Nigeria, on the other hand, has established a national disaster risk management policy, a national policy on internally displaced persons, and a specialized statutory body—the National Commission for Refugees, Migrants, and Internally Displaced Persons (NCFRMI)—to coordinate protection and durable solutions. The lack of a policy framework and weak human rights mechanisms for IDPs in India leaves them less protected and more vulnerable to the health challenges of TB, and this further increases their risk of poor health outcomes.

### 3.3 Intervention Strategies to Barriers in TB Care Among IDPs

#### 3.3.1 Sociocultural

India has a National Framework for a Gender‐Responsive Approach to TB [43]. The framework is based on the “principles of non‐discrimination, informed choice, informed consent, confidentiality, respect for all, access for all, working in partnership, promoting rights of individuals and groups, fostering accountability and empowering communities.” The sections of the NTEP framework include “Detect,” “Treat,” “Prevent,” and “Build.” The framework addresses gender‐based issues that affect TB treatment adherence by training healthcare workers, building the capacity of non‐governmental partners, and empowering women and men in the TB response at the community and health system levels [43]. Action Against Stigma (AAS) is an initiative of the TB association of India that is addressing myths and misinformation about the causes and effects of TB through community‐based awareness programs. Some of the myths about TB that AAS has addressed include “TB is hereditary,” “TB affects only the poor,” “TB treatment is ineffective,” and “TB patients should be completely isolated” [44].

#### 3.3.2 Economic

The India NTEP, administered by the Ministry of Health and Family Welfare, Government of India, provides free TB screening, diagnosis, and treatment for all documented TB cases [45]. This provision of free screening services is a key step in breaking the financial barrier to TB care. India operates the *Ni‐kshay Poshan Yojana* (NPY). This is a direct cash transfer (also known as direct benefit transfer) scheme under the NTEP. The NPY offers a monthly cash benefit of INR 500 to all notified patients with TB for nutritional support during the period of anti‐TB treatment [46]. This social protection intervention, integrated into national‐level TB control programs, addresses needs such as transportation and nutritional support for TB cases and their households [46]. This initiative has contributed to improved food security, enhanced TB treatment adherence, and better treatment outcomes.

#### 3.3.3 Health Governance and Policy

India is making progress in strengthening systems for improved TB case detection, diagnosis, treatment, and prevention. In 2020, the Revised National Tuberculosis Control Program (RNTCP) was renamed the NTEP. This aligns with India's commitment to eliminate TB by 2025, just 5 years ahead of the global target of 2030. Some of the strategies in the reformed RNTCP include multi‐sectoral partnerships and collaborations with other institutions, such as nonprofit organizations, strengthening the patient support system, implementing house‐to‐house screening to increase detection, and working with 560 colleges to support TB detection and research [46]. There is no known or published evidence that India currently mobilizes funding for the building of health clinics in displacement camps with the supply of quality medicines, including anti‐TB treatment. However, India has an NTEP with key strategies aimed at achieving the elimination of the disease by 2030 [47].

India has demonstrated effective leadership in driving its NETP toward its national strategic goal. It has demonstrated strong political will and commitment, which has cascaded from the strategic to the operational level across its administrative arms of health governance [47, 48]. Key initiatives and strategies driven by effective governance of the TB prevention and control infrastructure include decentralizing TB interventions through active stakeholder participation at local and community levels and leveraging innovative technologies, including digitization, to accelerate the TB response. Additionally, strong leadership has led to the development of improved TB surveillance systems, supported by artificial intelligence, enhanced TB case reporting, and effective communication with the public through TB helplines and call centers [47, 48]. As part of its NTEP, India has decentralized TB services with the village‐free TB strategy. The Pradhan TB Bharat Abhiyan initiative is community‐driven and aims to involve citizens through active participation in TB elimination efforts [48].

## 4 Discussion

The review offers a comparison of the contextual barriers to TB care access by a less studied vulnerable population in India and Nigeria. It also examined country‐specific interventions relevant to expanding TB care access. The burden of TB in India and Nigeria reflects the proportion of material, human, and logistic resources required by national control programs to address the threat [49]. Given finite resources, intersectoral collaboration is essential to address barriers to TB care access among internally displaced persons [50]. Nonprofit and faith‐based organizations can collaborate with the government and other private‐sector partners to expand screening, health education, nutrition, and treatment interventions in displaced camps [51, 52]. TB interventions are not standalone as they require integration with other efforts in nutrition, finance, and infection control for lasting impact [53]. Although respective national governments make steady progress in innovative approaches to TB prevention and control, expanding TB care access to IDPs should not only be about improving haphazard screening and diagnostic infrastructure but also through the establishment of strong healthcare reforms that are based on quality research and backed by public policy that is rooted in principles of health equity and justice [54]. These reforms can strengthen the collection of TB‐related data in IDPs living with TB, characterize the internally displaced population to enhance TB surveillance, and plan for resource allocation.

National TB control programs in India and Nigeria operate strategically to achieve national benchmarks in TB prevention and control. However, the strategies used by these national programs must be comprehensive, inclusive, and adapted to the needs of internally displaced persons. The following recommendations are made to improve programmatic response to TB among internally displaced persons.

1. **Integrated healthcare delivery**: TB control activities should be integrated into primary healthcare services within IDP settings, rather than maintaining parallel or fragmented systems. This will promote cost‐saving and efficiency in TB case detection and treatment.
2. **Improve pathway to treatment entry**: Active case finding should be improved through routine symptom screening, establish reliable sputum collection and GeneXpert referral networks, and enable prompt treatment initiation within camps where feasible.
3. **Geographic accessibility**: Mobile and outreach TB services are essential programmatic tools for reaching displaced populations affected by geographic isolation, transport barriers, and weak facility access, with deployment adapted to camp movement patterns and security conditions.
4. **Multi‐contextual intervention mechanisms**: Effective control of TB in the IDP context requires programs that are designed to address structural and social determinants of disease. This includes gender‐responsive TB programming that reduces access barriers for women, strengthens confidentiality, and engages household decision‐makers in care‐seeking processes. TB control programs must also incorporate financial and nutritional support mechanisms, such as transport assistance, cash transfers, and food supplementation, to reduce catastrophic costs and address TB–malnutrition interactions.
5. **Decentralized surveillance systems**: Improving data disaggregation for IDPs and ensuring their explicit inclusion in national TB control strategies are essential for accountability and planning.

## 5 Conclusion

As both high TB burden countries in Africa and Asia, respectively, Nigeria and India share demographic, cultural, and socioeconomic characteristics. The internally displaced populations of both countries are ethnically different, but they share similar hopes and aspirations—the removal of barriers to quality TB care, including treatment and preventive services. India is implementing strategies and programs like those recommended for Nigeria. It also has a national TB control program, like Nigeria's, but lack of a national policy framework and human rights mechanisms to address the needs of internally displaced persons sets both countries apart from each other [54]. Overall, effective TB control among IDPs requires integrated service delivery, multi‐sectoral support, and alignment with humanitarian health and human rights frameworks.

## Author Contributions

**Linnaya Graf**: writing – review and editing, conceptualization, methodology. **Paul West Okojie**: conceptualization, methodology, writing – review and editing, writing – original draft, formal analysis.

## Funding

The authors have nothing to report.

## Conflicts of Interest

The authors declare no conflicts of interest.

## Supporting information




**Table S1:** Detailed search strategies used across electronic databases.

## Data Availability

Data sharing is not applicable to this article as no datasets were generated or analyzed during the current study.
