# Supplementary material for: Tuberculosis Care Access Among Internally Displaced Persons in India and Nigeria: A Review of Structural Barriers and Context‐Specific Interventions
Source: Public Health Chall. 2026 Jul 23;5(3):e70319. doi: 10.1002/puh2.70319 (PMC13394371; doi:10.1002/puh2.70319)
Supplement: Supplementary file 1 — Table S1: Detailed search strategies used across electronic databases. [file PUH2-5-e70319-s001.docx]

| **Database** | | **Search strategy** |  | |
| --- | --- | --- | --- | --- |
| PubMed | \|  \| \| --- \|   ("Tuberculosis"[MeSH] OR tuberculosis OR TB OR "pulmonary tuberculosis" OR "drug-resistant tuberculosis" OR MDR-TB OR XDR-TB) AND ("Refugees"[MeSH] OR "Transients and Migrants"[MeSH] OR "internally displaced" OR "internal displacement" OR IDP* OR "forced migration" OR "forcibly displaced" OR "displaced person*" OR "displaced population*" OR "conflict-affected" OR "humanitarian setting*" OR "armed conflict" OR insurgency OR refugee*) AND ("Health Services Accessibility"[MeSH] OR "Healthcare Disparities"[MeSH] OR access OR accessibility OR utilization OR "treatment access" OR "care access" OR "service delivery" OR coverage OR adherence OR "treatment outcome*" OR barrier* OR "structural barrier*" OR "health system*" OR intervention* OR program* OR strateg*) AND (India OR Nigeria) AND publication dates 2015–2025 AND English language. | | |  |
| Scopus | TITLE-ABS-KEY (tuberculosis OR TB OR "pulmonary tuberculosis" OR "extrapulmonary tuberculosis" OR "drug-resistant tuberculosis" OR MDR-TB OR XDR-TB) AND TITLE-ABS-KEY ("internally displaced" OR "forced displacement" OR IDP* OR "displaced population*" OR "forced migration" OR "conflict-affected" OR "humanitarian crisis" OR "armed conflict" OR insurgency OR refugee*) AND TITLE-ABS-KEY (access OR accessibility OR utilization OR "treatment access" OR "care access" OR coverage OR adherence OR "treatment outcomes" OR barriers OR "health systems" OR interventions OR programs OR strategies) AND TITLE-ABS-KEY (India OR Nigeria OR Borno OR Adamawa OR Yobe OR Kashmir OR Assam OR Manipur) AND publication years 2015–2025 AND English language. | | |  |
| Global Index Medicus | Separate searches conducted using the terms: "tuberculosis displaced"; "tuberculosis IDP"; "tuberculosis conflict"; "tuberculosis insurgency"; "tuberculosis humanitarian"; "tuberculosis refugee"; "tuberculosis migrant"; "TB displaced"; "TB conflict"; "tuberculosis Borno"; and "tuberculosis Kashmir". Results from all searches were combined and deduplicated. | | |  |
| Google Scholar | \|  \| \| --- \|   Separate searches conducted using the terms: "tuberculosis displaced"; "tuberculosis IDP"; "tuberculosis conflict"; "tuberculosis insurgency"; "tuberculosis humanitarian"; "tuberculosis refugee"; "tuberculosis migrant"; "TB displaced"; "TB conflict"; "tuberculosis Borno"; and "tuberculosis Kashmir". Results from all searches were combined and deduplicated. | | |  |
| Grey Literature | Site-restricted Google searches of WHO, UNHCR, IOM, ReliefWeb, Stop TB Partnership, TBFacts, Government of India, and Nigeria CDC websites using combinations of tuberculosis/TB with displacement, internally displaced persons, conflict, migration, access, and Nigeria/India. Additional targeted searches and manual review of WHO Global TB Reports, WHO IRIS, UNHCR Operational Data Portal, IOM DTM reports, ReliefWeb, NTEP reports, and NTBLCP reports. | | |  |

**Table S 1.** Detailed Search Strategies Used Across Electronic Databases
